# Localised pericardial tamponade diagnosed by computed tomography: a case presentation

**DOI:** 10.1186/1752-1947-1-162

**Published:** 2007-12-01

**Authors:** Hunaid A Vohra, Hazem Khout, Deepashree Bapu, Qamar Abid

**Affiliations:** 1 Department of Cardiothoracic Surgery, University Hospital of North Staffordshire NHS Trust, Stoke-on-Trent, UK; 2 Department of Cardiac Surgery, Harefield Hospital, Royal Brompton & Harefield Hospitals NHS Trust, London, UK

## Introduction

In a normovolemic patient, low cardiac output after cardiac surgery may be a result of myocardial ischaemia and/or pericardial tamponade. However, without any objective evidence of ischaemia alongwith no signs of pericardial tamponade or regional wall motion abnormality on transthoracic echocardiogram (TTE), the diagnosis remains ambiguous. Computed tomography (CT scan) of the chest may be helpful to reveal pericardial tamponade.

## Case presentation

A 73 year old, hypertensive and hypercholestremic gentleman, presented to the Emergency Department with acute onset of severe retrosternal chest pain. He had no other significant co-morbidities. ECG showed ST segment depression in leads I, AVL, V5 and V6. The troponin I level was 4.1 ng/ml. A diagnosis of non-ST elevation myocardial infarction (NSTEMI) was made. The patient was given aspirin, clopidogrel and subcutaneous clexane. During the admission he continued to get chest pain intermittently, which required intravenous glyceryl trinitrate infusion. A coronary angiogram was performed 4 days later, which revealed significant stenosis of the proximal left anterior descending artery (LAD) and circumflex artery (Cx) as well as an occluded right coronary artery (RCA) in the mid-vessel. A TTE showed moderately impaired left ventricular ejection fraction (<50%) He was referred for urgent coronary artery bypass grafting (CABG) which he underwent a week after admission. The operation was performed via a sternotomy under cardiopulmonary bypass (CPB) with aorto-atrial cannulation and antegrade cold blood cardioplegia. The patient was cooled to 32°C. The left internal mammary artery was anastomosed to the LAD, reversed long saphenous vein (LSV) grafts were performed to posterior descending artery and left ventricular branch of RCA as well as obtuse marginal and diagonal arteries (CABG times 5). The CPB time was 85 minutes and the cross-clamp time was 65 minutes. The heart was weaned off CPB easily without any inotropes. A left pleural and mediastinal drain was inserted. Following closure of the chest, he was transferred to the intensive care unit (ICU), where he made excellent progress initially and was extubated within 12 hours. At 24 hours post-operatively, the blood pressure (BP) was 110/85 mm Hg, the cardiac index (CI) was 3.0 litres/min/m^2 ^and the total amount of blood in the drains was 1350 mls, with no drainage in the last 2 hours. Within 2 hours of removing the drains, the BP dropped to 80/40 mmHg with a CI of 1.8 litres/min/m^2 ^with no change in the central venous pressure (CVP, 10 mm Hg), whilst the urine output was maintained at >0.5 ml/kg/hr. The systemic vascular resistance was 1150 dynes/cm^5^. No new changes were seen in the ECG.

**Figure 1:**
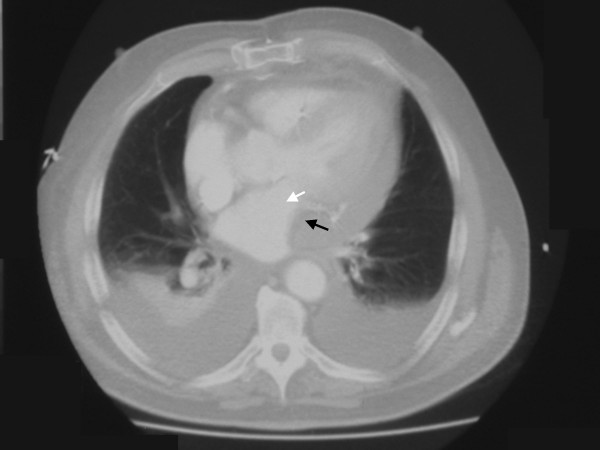
CT scan of the chest showing a large localised blood clot (black arrow) compressing the left ventricle (white arrow). Also note bilateral pleural effusions.

A TTE was performed by an experienced sonographer which showed similar left ventricular function as before and no evidence of pericardial collection or tamponade. In view of depressed LV function, 0.05 mcg/kg/min of adrenaline infusion was commenced and an intra-aortic balloon pump (IABP) was inserted in the right common femoral artery. Despite these measures, the CI index improved only to 2.0 litres/min/m^2^. By this stage, the CVP was 16 mmHg, the serum lactate increased from 1.0 to 4.1 and the urine output was 30 mls/hr. Despite a normal TTE, a strong suspicion of pericardial tamponade was made. A trans-oesophageal echocardiogram (TOE) was not available and it was decided to perform a CT scan of the chest (without contrast). A Siemens SOMATOM Sensation 16 slice CT scanner (Siemens Medical Solutions Inc, PA, USA) was used. Figure1 shows a localised 4 cm pericardial collection (black arrow) around the free wall of the left ventricle (white arrow) causing tamponade. Surgical exploration was contemplated. On removal of the wires at reopening, blood was released from the pericardium with pressure and large amount of clots were removed from around the LV. Thereafter, the BP improved to 125/85 mmHg with a CI of 4.3 litres/min/m^2^. The IABP was removed after 24 hours and the inotropes were weaned off. Thereafter, the patient made an unremarkable recovery and was discharged home on day 7.

## Discussion

Pericardial tamponade within the first few hours of cardiac surgery may lead to cardiac arrest. In the literature, the reported incidence is 0.2%–1.8%. [1,2]. In the majority of the patients (66%) who develop pericardial tamponade after cardiac surgery, pericardial collections located posteriorly are mainly responsible for haemodynamic instability while in the remaining one-third, collections around the right atrium and/or right ventricle are the cause [3]. The decision to re-explore the chest should be based on clinical suspicion derived from signs which include rising jugular venous pressure (CVP in monitored patients in ICU), low BP, muffled heart sounds (Beck's triad), narrowed pulse pressure, oliguria, low cardiac output and metabolic acidosis. However, if localised, pericardial tamponade may not manifest itself in the classical fashion and may be difficult to diagnose, even with TTE, especially when other causes of low cardiac output cannot be excluded.

It has been reported [4] that echocardiographic features like early diastolic RV collapse, RA collapse (which is more sensitive but less specific than RV collapse), left atrium (LA) collapse and phasic respiratory changes in RV and LV are useful signs of pericardial tamponade. However, if diastolic pressure is high in a cardiac chamber as a result of ventricular dysfunction or severely hypertrophied ventricle, then the classical echocardiographic signs of cardiac tamponade may not be visualised. Since, the features of ventricular dysfunction, hypertrophy and pulmonary hypertension are not uncommon in patients undergoing cardiac surgery, the commonly seen echocardiographic features of tamponade may be absent, even in severe tamponade. The finding of large respiratory fluctuations in the ventricular size due to bulging of the ventricular septum towards the LV with inspiration may also be masked with septal hypertrophy. Oyama et al [5] have discussed the usefulness of CT in the detection of pericardial effusions. While simple pericardial effusions have attenuation of water, attenuation greater than water is highly suggestive of haemopericardium in the post-cardiac surgery setting. Furthermore, CT scan can visualise the whole of the thoracic cavity whereas echocardiography shows limited views. Sonolucent areas adjacent to the pericardium like pleural effusions and pericardial cysts can sometimes be mistaken for pericardial collections by echocardiographers but this can be clearly differentiated with CT scan. Although, detection of retrosternal localised post-cardiac surgery effusions with echocardiography has been reported [6], this is considered to be a very difficult area to examine in post-surgery patients, where anatomy is distorted. In another case report [7], in the setting of penetrating thoracic trauma, the echocardiographic findings were inconclusive and contrast-enhanced computed tomography (CT) with fine reconstructions was performed which enabled the authors to reach a diagnosis of right ventricular rupture leading to pericardial tamponade.

## Conclusion

There is no doubt that a low cardiac output after CABG should immediately draw attention towards pericardial tamponade. Indeed, pericardial tamponade is a clinical diagnosis. However in cases where clinical diagnosis is inconclusive, echocardiography may be helpful. Echocardiography, despite being considered the gold standard investigation for detecting cardiac tamponade, may be unhelpful in certain cases and a consensus to re-explore may not be achieved. In case of strong clinical suspicion and negative echocardiographic findings, we suggest that alternative modalities like CT scan may prove to be invaluable to reach a surgical decision.

## Competing interests

The author(s) declare that they have no competing interests.

## Authors' contributions

HAV- major contribution to the writing of the paper and collection of clinical material

HK- collection of clinical material and writing of paper

DB- writing of paper

QA- writng of paper and final approval

## Consent

Patient consent was received for the manuscript to be published.
